# Ultrafast sound production mechanism in one of the smallest vertebrates

**DOI:** 10.1073/pnas.2314017121

**Published:** 2024-02-26

**Authors:** Verity A. N. O. Cook, Antonia H. Groneberg, Maximilian Hoffmann, Mykola Kadobianskyi, Johannes Veith, Lisanne Schulze, Jörg Henninger, Ralf Britz, Benjamin Judkewitz

**Affiliations:** ^a^Einstein Center for Neuroscience, Charité Universitätsmedizin Berlin, Berlin 10117, Germany; ^b^Department of Biology, Humboldt University, Berlin 10115, Germany; ^c^Senckenberg Society Natural History Collections, Dresden 01109, Germany

**Keywords:** animal communication, sound production, biomechanics, optical transparency, emerging model system

## Abstract

Motion is the basis of nearly all animal behavior. Evolution has led to some extraordinary specializations of propulsion mechanisms among invertebrates, including the mandibles of the dracula ant and the claw of the pistol shrimp. In contrast, vertebrate skeletal movement is considered to be limited by the speed of muscle, saturating around 250 Hz. Here, we describe the unique propulsion mechanism by which *Danionella cerebrum*, a miniature cyprinid fish of only 12 mm length, produces high amplitude sounds exceeding 140 dB (re. 1 µPa, at a distance of one body length). Using a combination of high-speed video, micro-computed tomography (micro-CT), RNA profiling, and finite difference simulations, we found that *D. cerebrum* employ a unique sound production mechanism that involves a drumming cartilage, a specialized rib, and a dedicated muscle adapted for low fatigue. This apparatus accelerates the drumming cartilage at over 2,000 g, shooting it at the swim bladder to generate a rapid, loud pulse. These pulses are chained together to make calls with either bilaterally alternating or unilateral muscle contractions. *D. cerebrum* use this remarkable mechanism for acoustic communication with conspecifics.

## Significance

Due to its small size and lifelong optical transparency, the fish *Danionella cerebrum* is an emerging model organism in biomedical research. How can this small vertebrate under 12 mm length produce sounds over 140 dB? We found that it possesses a unique sound production apparatus – involving a drumming cartilage, specialized rib, and fatigue-resistant muscle – which allows the fish to accelerate the drumming cartilage at extreme forces and generate rapid, loud pulses. Our finding challenges the conventional notion that the speed of vertebrate skeletal movement is limited by muscle action. Understanding this extraordinary adaptation expands our knowledge of animal motion and highlights the remarkable diversity of propulsion mechanisms across species, contributing to our broader understanding of evolutionary biology and biomechanics.

Many animals use acoustic signals to communicate (1). Although the mechanism for sound production varies across vertebrates, it is common to use specialized striated muscles that manipulate air (2). In tetrapods, sounds are controlled by vocal folds in the larynx (3) or the syrinx in birds (4), which modulate airflow. In these cases, sound production and respiration are coupled, but fishes, with some exceptions (5), do not breathe air; therefore, the mechanisms they use for sound production are not coupled with airflow and they instead rely on other mechanisms (2).

The most thoroughly described sound production mechanism in fishes involves vibrations of the swim bladder driven by rhythmic contractions of specialized “sonic” or “drumming” muscles (6). The swim bladder is a gas-filled cavity, predominantly used to control buoyancy. Drumming muscles can be either directly attached to the swim bladder or coupled to the swim bladder indirectly via other structures such as ligaments or bony plates (7). Typically, swim bladder mechanisms involving contractions of direct sonic muscles produce tonal sounds with fundamental frequencies on the order of 100 Hz, such as in the plainfin midshipman (8) and the oyster toadfish (9). This frequency is determined by the contraction rate of the sonic muscle (10) as the muscle directly drives the oscillation.

Bony fishes can also use stridulation to produce sounds. In this case, pulses are generated by skeletal elements such as teeth, fin rays, or vertebrae rubbing together (11). Typically, the pulses are produced rapidly but with irregular intervals, which creates chirping or raspy sounds. These pulses have a wide frequency spectrum with a non-harmonic structure (12) and have dominant frequencies on the order of 1 kHz which is higher than the sounds produced by swim bladder mechanisms (13).

**Fig. 1.**
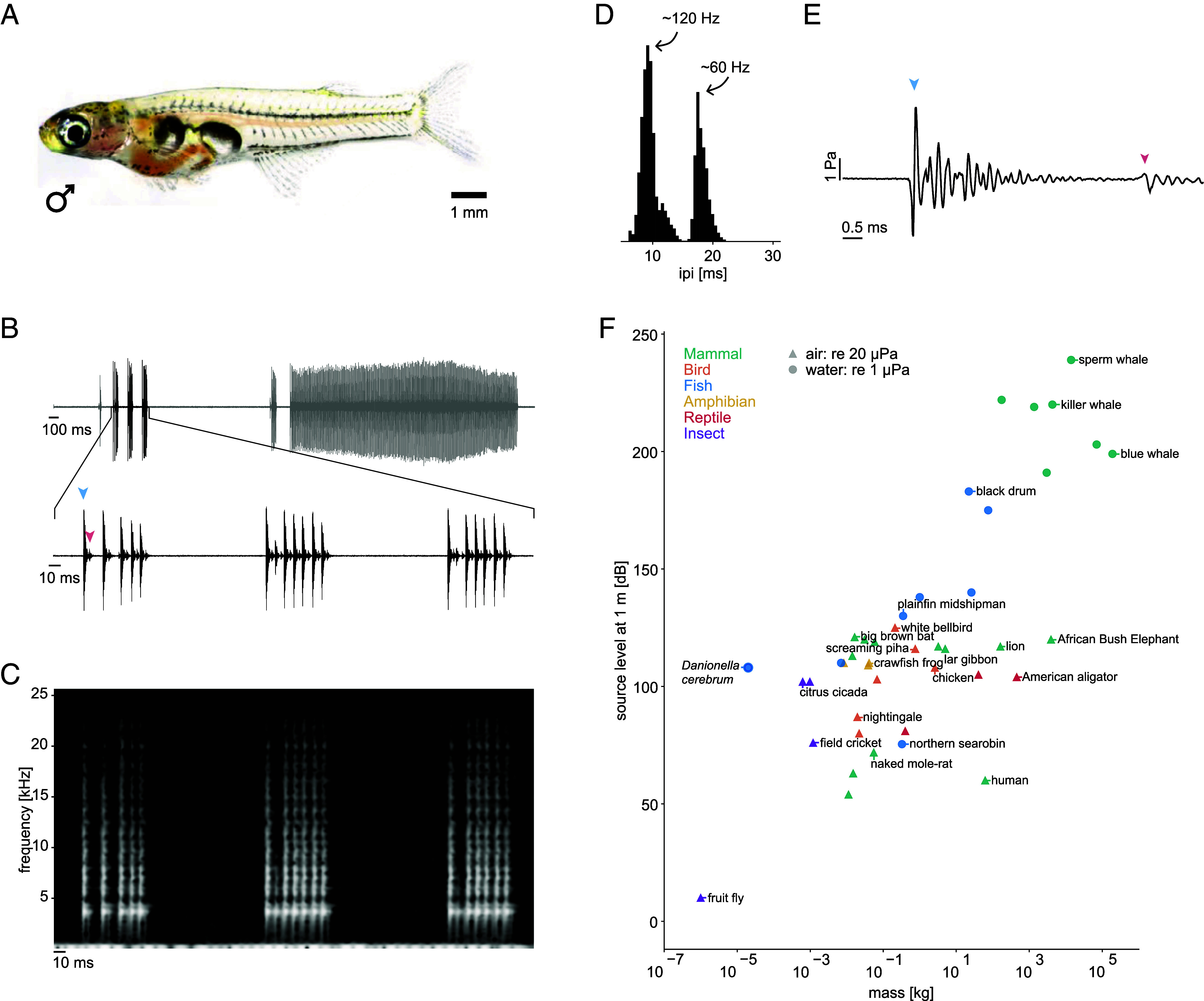
*Danionella cerebrum* vocalizations. (*A*) Photo of a *D. cerebrum* male. Note the distinct separation between the anterior and posterior swim bladder chambers. (*B*) Example of vocalizations (Audio S2). *D. cerebrum* produce pulses that are arranged into bursts composed of up to several hundred pulses (*Top*). Zoom-in of the audio trace (*Bottom*) shows that the large pulse (blue arrow head) is followed by a quieter after-pulse (red arrow head). (*C*) Spectrogram of the audio trace shown at the *Bottom* of (*B*). The pulses are broadband and have a dominant frequency component around 5 kHz. (*D*) A histogram of the inter-pulse interval has a bimodal distribution with peaks at 9.25 ± 0.97 and 17.98 ± 0.96 ms, estimated from the mean and SD of a Gaussian fit. This corresponds to rates of around 120 and 60 Hz, respectively. (*E*) Waveform of a single pulse. The main pulse (blue arrow head) begins with a rarefaction and completely decays before the after-pulse (red arrow head) starts. The amplitude of a single pulse can reach 7 Pa, corresponding to 137 dB (re 1 μP). The oscillations after the initial pulse are likely due to reflections (*SI Appendix*, Fig. S1). (*F*) Relative to their body weight, *Danionella* (blue dot) sounds are particularly loud compared with those of communication calls in other animals. All dB values are given relative to the standard reference pressures of 1 µPa in water and 20 µPa in air.

In *Danionella cerebrum*, a miniature teleost with the smallest known vertebrate brain (14–17) (Fig. 1*A*), the males can produce sounds (Fig. 1*B*) (Audios S1 and S2) in social contexts (Movie S1). Their vocalizations (18, 19) are composed of discrete pulses with a broad frequency spectrum exceeding 20 kHz (14) (Fig. 1*C*). They are around 2.5 ms in duration and are followed by a quieter “after-pulse” (Fig. 1 *B* and *E*). The pulses typically appear in repetitive sequences of variable duration (hereafter referred to as bursts). The time intervals between pulses in a burst have a bimodal distribution with pulse rates of either ~120 Hz or ~60 Hz (Fig. 1*D*).

The sound production mechanism of the pulses generated by *D. cerebrum* has been a mystery, as neither stridulatory nor swim bladder-related muscular mechanisms seem to provide plausible explanations for the origin of the sound. The broad frequency profile and short duration of the pulses suggest stridulations, but the regularity of the inter-pulse interval contradicts this hypothesis. The alternative hypothesis of a conventional swim bladder mechanism appears to be equally implausible because it is unclear how it would create brief pulses, rather than harmonic tones, at this rate—seemingly ruling out the two most common mechanisms for sound production in fishes.

Anatomical studies of the closely related species *Danionella dracula* have revealed distinct structures adjacent to the swim bladder (20), including a bulbous muscle, hypertrophied rib, and globular cartilage. It has been hypothesized that sounds may be generated when the cartilage hits the swim bladder (20). However, this hypothesis has not been confirmed by in vivo recordings from an intact animal and it is unclear how these components would interact with each other.

In this study, we used high-speed video recordings to investigate the mechanism of sound production. In addition, we used micro-CT scans to reveal the components of the sonic organ in *D. cerebrum* in combination with differential gene expression to identify its specialization. From this data, we constructed a model of the sound production mechanism employed by *D. cerebrum* males.

## Results

To characterize the intensity of *D. cerebrum* vocalizations, we measured the pulse amplitude. The fish were placed in a large tank, to reduce the effects of echoes from the aquarium walls, and hydrophones were spaced equally around the center (*Methods*). Although the fish are only 10 to 12 mm long, the amplitude of a single pulse could reach pressure levels of more than 135 dB at a distance of 35 mm (Fig. 1*E*), corresponding to an amplitude of approximately 147 dB at 1 body length. Such an amplitude is highly unusual for an animal of its size (Fig. 1*F* and Dataset S1).

### High-Speed Video Recordings Reveal Swim Bladder Sound Production Mechanism.

**Fig. 2.**
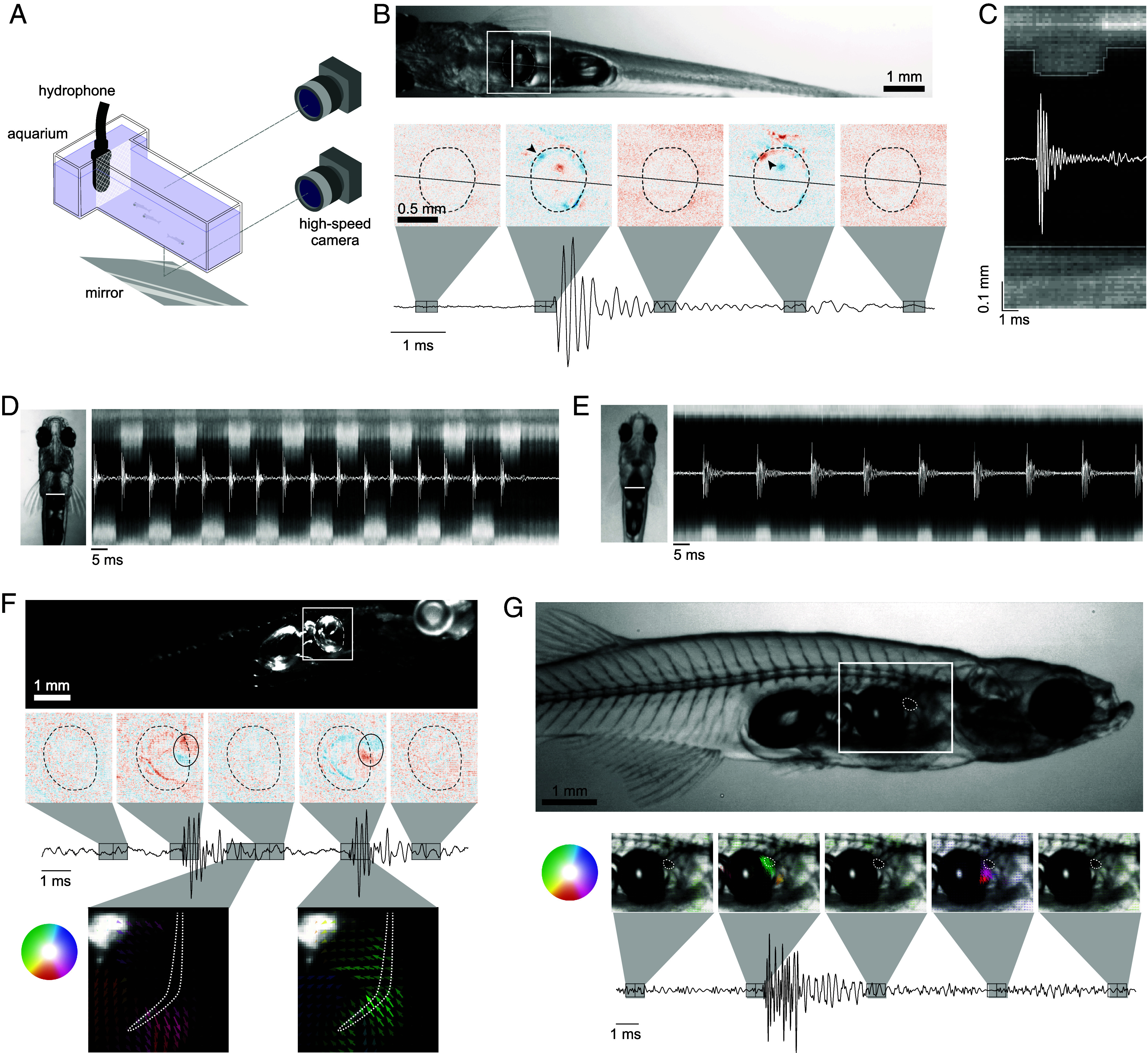
High-speed video reveals ultrafast motion during sound production. (*A*) High-speed video setup. 3 to 4 fish, including at least one male were placed inside the tank, separated from the hydrophone by a piece of mesh. The camera was moved to image the tank either from the side or from below with the use of a mirror. (*B*) A video of a male (Movie S2) (example frame above) was registered and the difference between consecutive frames displays the unilateral compression and relaxation of the anterior part of the swim bladder (black arrow heads) which corresponded with the onset of the pulse and after-pulse, respectively. (*C*) The pixels along the vertical white line in *B* were plotted over one pulse to show the dynamics of the swim bladder during sound production. The compression occurred over less than 0.125 ms (faster than one frame) whereas the relaxation occurred over about 0.5 ms. (*D*) A high-speed video from below (Movie S5) was registered and the pixels along the vertical white line in the example frame were plotted over a burst with a pulse rate of 120 Hz. The compression of the swim bladder alternated between the left and right side of the fish. (*E*) The same as in (*D*) except for a 60 Hz burst (Movie S6). The swim bladder compression is unilateral in this case. (*F*) A video of a male viewed from the side (Movie S7) (example frame above) with the illumination from above was registered and the difference between consecutive frames revealed that only a small area of the anterior part of the swim bladder was compressed, outlined with a black oval. In this view, we also observed rostral movement of the rib before the pulse (*Bottom Left*), and caudal movement after (*Bottom Right*). (*G*) A video of a male from the side (Movie S8) (example frame above), with backlight illumination. The optical flow between consecutive frames shows the motion of the cartilage, outlined with a white dotted line. The color and opacity of the arrows corresponds to the direction and amplitude of the motion respectively.

To identify the mechanism of sound production, groups of 3 to 4 fish, including at least one male, were placed in a small aquarium (9 × 6 × 2 cm) illuminated from above or from the side with an infra-red LED (Fig. 2*A*). Pulses produced by the fish triggered the recording of high-speed videos at selected frame rates between 2,000 and 8,000 fps.

Viewing the fish under bright field illumination (Fig. 2*B* and Movies S2 and S3), we observed that the compression of the frontal part of one side of the anterior swim bladder coincided with the onset of a single pulse. The oscillations of the pulse decayed while the swim bladder was still compressed, and the relaxation of the swim bladder correlated with the quieter after-pulse (Fig. 2 *B* and *C*). By modeling the compression of the swim bladder with a finite difference simulation, the oscillations in the audio trace after the initial pulse can be explained by echoes from the walls of the tank (*SI Appendix*, Fig. S1 and Movie S4).

For pulse bursts at 120 Hz, the compression of the swim bladder alternated between the left and right sides, each at a rate of 60 Hz (Fig. 2*D* and Movie S5). Vocalizations with a pulse rate of 60 Hz were created with unilateral compressions repeated on the same side of the body (Fig. 2*E* and Movie S6). No other fish has been reported to use repeated unilateral muscle contractions for sound production.

Each individual pulse occurred extremely rapidly, with the compression of the swim bladder happening over just one frame of a video recorded at 8,000 fps (Fig. 2*C*), implying an action faster than 125 µs. The swim bladder is compressed by ~150 µm, and assuming constant acceleration over this distance, the acceleration of the swim bladder wall has a lower bound of around 20,000 ms^−2^. Modeling the swim bladder as a monopole sound source, this compression was calculated to result in a sound pressure level (SPL) of 93 to 112 dB re 1 μPa at 1 m (*SI Appendix*, Fig. S2), consistent with the measured amplitude (Fig. 1*F*). The relaxation of the swim bladder was slower, occurring over 0.4 to 0.6 ms, which would explain the lower amplitude of the after-pulse. These movements are over one order of magnitude faster than any known muscle can contract (21). This ultrafast compression of the swim bladder indicates that *D. cerebrum* do not use muscle contractions to directly vibrate the swim bladder, suggesting the involvement of other structures.

Viewing the fish under dark-field illumination, we observed that only a small area of the swim bladder was compressed (Fig. 2*F* and Movie S7). In this lighting arrangement, the fifth rib that lies adjacent to the swim bladder was illuminated. It moved rostrally in the lead-up to the pulse, and then stopped when the pulse was produced, before returning to its original position. During the times when the fish was not vocalizing, the rib remained stationary in its resting position. This rib is sexually dimorphic and is larger and heavily ossified in males compared to females (*SI Appendix*, Fig. S3).

When fish were backlit, we observed the movement of a dark structure, just anterior to the swim bladder, that correlated with pulse production (Fig. 2*G* and Movie S8). This structure moved rapidly toward the swim bladder at the onset of a pulse, and away from the swim bladder at the onset of the after-pulse. Based on previous studies on *Danionella* anatomy (16, 20) and cartilage stains of *D. cerebrum* (*SI Appendix*, Fig. S3), we inferred that this object is the globular drumming cartilage, approximately 250 µm in length.

Therefore, our high-speed video recordings have revealed that *D. cerebrum* use a unique mechanism involving unilateral and bilateral alternating compressions of the swim bladder where the compressions are extremely fast and that the rib and cartilage motion correlates with the pulse production.

### Sound Production Apparatus in *D. cerebrum*.

**Fig. 3.**
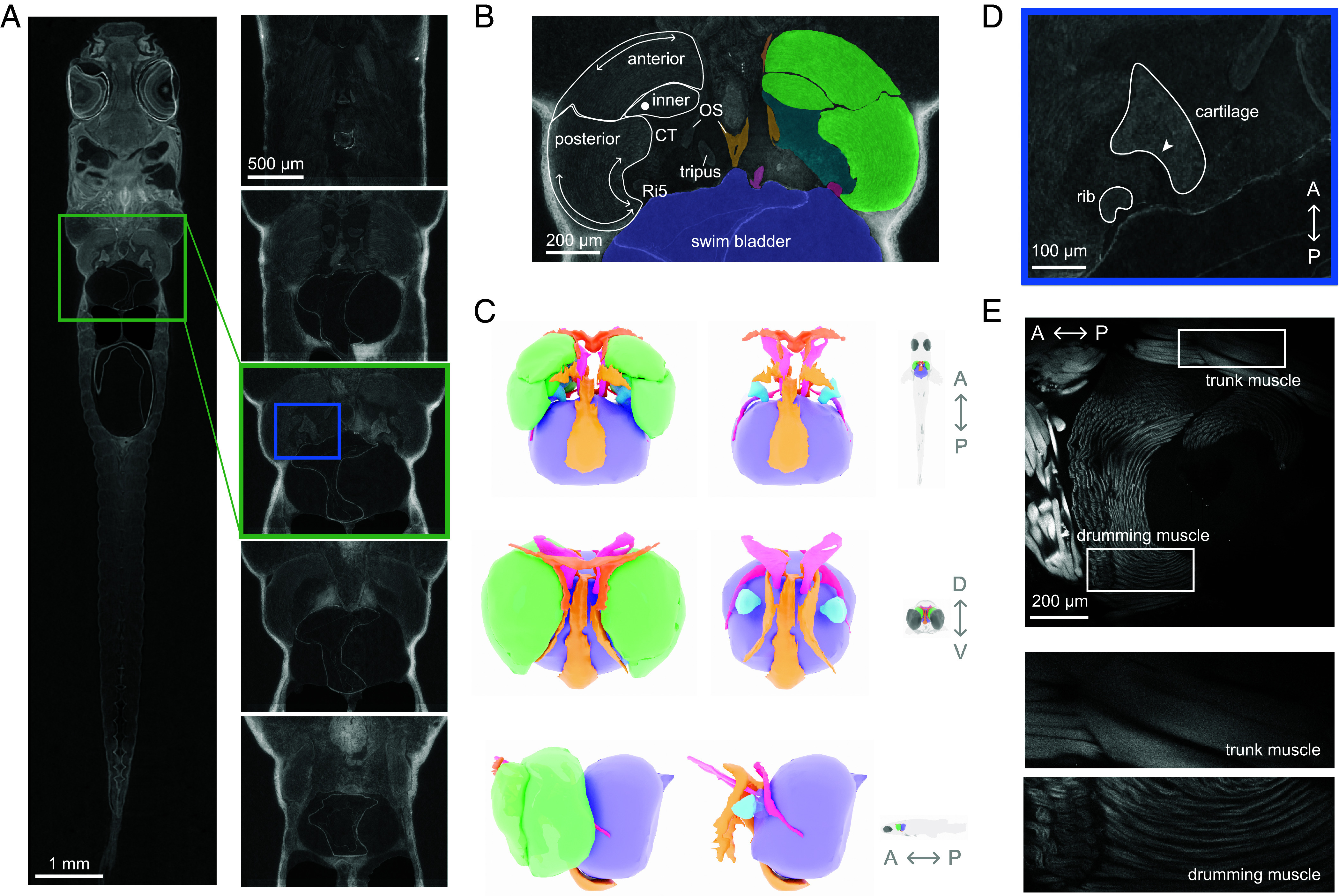
Anatomy of *D. cerebrum* sonic organ. (*A*) Slice of the micro-CT scan (*Left*) with sections of the sonic organ (*Right*) from dorsal (*Top*) to ventral (*Bottom*). (*B*) The distinct parts of the drumming muscle are outlined on the *Left*, with arrows indicating the direction of the muscle fibers in each section. On the *Right*, the different components of the sonic organ are segmented. (*C*) A 3D reconstruction of the sonic organ from different views, and with the drumming muscle removed on the *Right* to reveal the cartilage. A, P, D, V refer to the axis of the fish: anterior, posterior, dorsal and ventral, respectively. (*D*) A close-up of the area in the box in the *Middle* CT slice in *A*. The rib fits into the groove in the cartilage, labeled with the arrowhead. (*E*) A phalloidin stain shows the difference in structure between the drumming muscle and the trunk musculature. The drumming muscle fibers are thinner and are arranged radially, whereas the trunk muscles run anteroposteriorly.

To investigate how the rib and drumming cartilage interact with other components of the drumming apparatus during sound production, we studied the morphology of *D. cerebrum* using micro-CT scans (Fig. 3*A*).

The pair of sonic muscles are located laterally to the anterior swim bladder but are not directly connected with it. Each sonic muscle is composed of 3 distinct parts with different muscle fiber directions: anterior, posterior, and inner (Fig. 3*B*). The anterior part of the muscle is connected to the third vertebra, whereas the posterior part originates on the outer arm of the *os suspensorium* and inserts on the tip of the fifth rib. The *os suspensorium* is a bony structure that extends ventrally from the fourth vertebra and cradles the swim bladder, which possibly helps with stabilization during vocalizations (*SI Appendix*, Fig. S3).

We segmented individual structures from the scan and reconstructed the sonic organ in order to have a more comprehensive view of the different components (Fig. 3*C*). The sonic muscle forms a medial cavity for the drumming cartilage which is held in place with strands of connective tissue (20). The rapid movement of the cartilage observed in the high-speed videos (Fig. 2*G* and Video S8) could not be explained by the contraction of the muscle alone as the fastest reported muscle, the sonic muscle of the oyster toadfish (21), contracts at a rate of only a few hundred Hz. We noticed that the fifth rib is positioned such that it fits into a groove of the cartilage (Fig. 3*D*). The movement of this rib observed in the high-speed videos (Fig. 2*F* and Movie S7) would result in contact between the rib and the cartilage. The muscle would have to be specialized in order to move the rib which exerts force on the cartilage and not fatigue when producing long bursts at a consistent pulse rate (Fig. 1*B*).

### Specialization of the Drumming Muscle.

To investigate the adaptations of the drumming muscle, we performed differential gene expression analysis to compare the sonic muscle with regular trunk musculature in the tail. We found that the gene-ontology categories associated with the genes that were overexpressed in the drumming muscle were related to mitochondria (*SI Appendix*, Fig. S4). Equivalently, of the genes associated with mitochondria that we identified, 94% were overexpressed in the drumming muscle with an average threefold change (factor 2^1.6 ± 0.9^). The increased mitochondria in the drumming muscle, compared to the trunk muscle, means that it would be more resistant to fatigue.

To investigate the microanatomy of the drumming muscle, we stained the actin filaments with phalloidin conjugated with Alexa Fluor 594 (Fig. 3*E*). The fibers of the anterior and posterior part of the muscle lay in the horizontal plane, whereas the fibers of the inner part of the muscle ran dorso-ventrally, perpendicular to the anterior and posterior part. The sonic muscle fibers had a smaller diameter than those of the trunk muscle, a feature that is typical of fast muscles (11). Taken together, these data suggest that the muscle is specialized to resist fatigue and has at least three parts with possibly different functional roles.

### A Catch-and-Release Mechanism Produces Rapid Movement of the Cartilage.

**Fig. 4.**
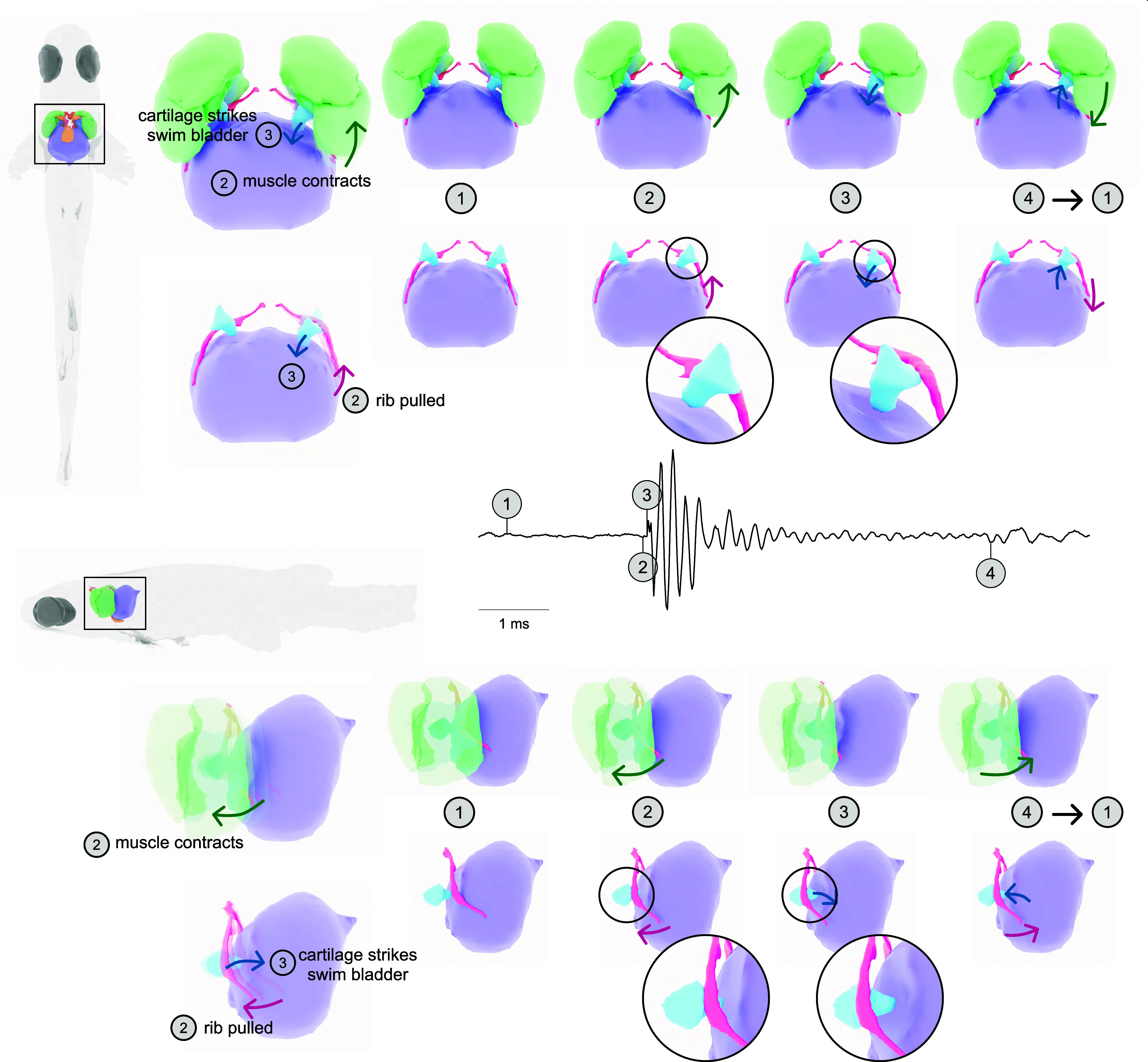
Catch-and-ultrafast release mechanism. The model we hypothesized for the sound production mechanism viewed from below (*Top*) and from the side (*Bottom*) for easier direct comparison with the high-speed videos. Before the pulse, the sonic organ is stationary (1). In the lead-up to the pulse production, one of the drumming muscles contracts, pulling the rib rostrally and the rib hooks into the groove in the cartilage, creating tension (2). The pulse is created when the tension is released as the cartilage rapidly strikes the swim bladder (3). Then, the muscle relaxes and the rib moves caudally, allowing the cartilage to return to its original position. As the cartilage moves back, the pressure in the swim bladder is relieved, producing the quiet after-pulse (4). The system then returns to its resting position (1) so the cycle can start again (Movie S9).

Combining the anatomical details with information from the high-speed videos allowed us to develop a dynamic three-dimensional (3D) model of the sound production mechanism in *D. cerebrum* (Fig. 4 and Movie S9). In the high-speed videos, the movement of the 5th rib correlated with pulse production (Fig. 2*D*). We hypothesize that the posterior part of the muscle contracts and pulls the rib anteriorly. The rib meets the groove in the cartilage (Fig. 3*D*) and tension is built as the rib pulls the cartilage along. The tension is suddenly released as the cartilage snaps out of the hold of the rib and it strikes the swim bladder (Fig. 2*E*). The swim bladder then acts as a heavily damped oscillator, producing a short, loud pulse. Then, the muscle relaxes, releasing the rib caudally and the connective tissue surrounding the cartilage helps to return the cartilage to its resting position. When the cartilage is released from the swim bladder, the quiet after-pulse is produced (Fig. 2*C*). The sonic organ returns to its resting position, allowing the cycle to restart.

## Discussion

We have described the catch-and-release mechanism used by *D. cerebrum* for sound production. *D. cerebrum* have a pair of extrinsic, indirect sonic muscles that house the drumming cartilage. When a sonic muscle contracts, the fifth rib is pulled anteriorly and builds tension as it pulls the cartilage. The sudden release of the cartilage causes it to rapidly strike the swim bladder, producing a short, loud pulse. A burst of pulses is generated with either bilaterally alternating or unilateral muscle contractions. In sum, this mechanism enables loud and stereotyped sounds that can be elicited in structured sequences, making it a unique solution for acoustic communication and ultrafast skeletal motion in vertebrates surpassing the limitation of muscle contraction speed.

### *D. cerebrum* Produce Loud Sounds for Their Size.

Compared to other vertebrates, *D. cerebrum* produce unusually loud calls relative to their body size. Generating high SPL would require high energy expenditure which is perhaps why *D. cerebrum* utilize short, discrete pulses; a train of pulses can have a high peak pressure, while the energy over time is low compared with a continuous call.

Some invertebrates have developed mechanisms to produce extremely loud sounds (22, 23) and rapid movement (24) despite their small size. However, vertebrate motion is typically considered to be limited by the muscle contraction rate (21).

### Specializations of the Sonic Muscle for Rapid Contractions.

The smaller diameter of the fibers in the drumming muscle compared to regular trunk muscle means that there is less distance for the oxygen and glucose to travel from the sarcoplasm to the myofibrils allowing for sustained rapid contractions of the muscle (25). The increased count of mitochondria in the drumming muscle also allows for large energy consumption with reduced fatigue. This results in the ability of the drumming muscle to perform quick and consistent contractions for seconds at a time which allows *D. cerebrum* to produce long vocalizations with pulse rates at 60 or 120 Hz.

It is common for super-fast muscles in sonic organs in other animals, including the type I male midshipman sonic muscles (26) and the bat larynx, to contain a higher density of mitochondria than in their corresponding skeletal muscles (21). The volume required for the increased mitochondria counts reduces the space left for contractile components, so these fast muscles compromise force for speed. This could explain why *D. cerebrum* do not use direct muscles to vibrate the swim bladder but instead use an indirect mechanism that can exert more force, creating a louder pulse.

### Catch-and-Release Mechanism Creates Loud, Broadband Pulses.

It is common in otophysan fishes to have two swim bladder chambers, where the anterior swim bladder is specialized for hearing and sound production. It’s possible that the distinct separation in the two swim bladder chambers in *D. cerebrum* was adapted for sound production (27). There is a variety in swim bladder mechanisms that use extrinsic, indirect sonic muscles, although they operate at lower frequency and amplitude. A large number of catfishes have a sonic muscle that inserts onto a bony plate, sometimes called an “elastic spring” or “ramus Mülleri” (28), that is itself connected to the swim bladder. The contraction of the muscle causes the swim bladder to extend, building tension. When the muscle relaxes, the tension in the elastic spring is released, causing the swim bladder to snap back to its resting position (29). The pulse repetition rate is around 100 Hz with dominant frequencies below 2 kHz.

Carapid fishes also have extrinsic sonic muscles and use a catch-and-release mechanism for sound production (30). However, in this case, a tendon hook attached to the end of the sonic muscle directly pulls on the swim bladder wall, with the release causing vibrations of the swim bladder. Like with *D. cerebrum*, the contraction rate of the muscle determined the inter-pulse interval rather than the frequency of the pulses themselves. The inter-pulse interval in these carapids was relatively long compared to *D. cerebrum*, with a contraction rate of only around 10 Hz and the dominant frequency in a single pulse was 340 Hz.

By using a catch-and-release mechanism, rather than direct sonic muscles, *D. cerebrum* can create sounds with dominant high-frequency components without having to contract their muscles extremely rapidly. Low-frequency sounds attenuate rapidly in the shallow waters that *D. cerebrum* live in (16). These high-frequency sounds would travel further and reach conspecifics that are further away where visibility is low.

### Alternating and Unilateral Contractions.

The only other reported fish with alternating sonic muscle contractions is the northern sea robin (31). The northern sea robin has intrinsic sonic muscles that directly vibrate the swim bladder. In *D. cerebrum*, unilateral as well as alternating muscle contractions are used to generate sounds. As far as we are aware, unilateral contractions for a swim bladder sound production mechanism have never been reported in any other fish species before. Swim bladder mechanisms utilize the movement of air for sound production, but unlike with tetrapods, the air-flow itself cannot be manipulated, limiting the variety of sounds that can be generated. By using alternating and unilateral, rather than synchronous muscle contractions, a greater diversity of sounds can be produced. Assuming that the unilateral contraction rate is limited to 60 Hz, alternating contractions doubles the achievable frequency. This would be a compromise on the amplitude of the sound in favor of the ability to communicate a greater diversity of information.

The channel catfish has been reported to use alternating pectoral fin movements to produce stridulation sounds (32). Some catfish had a preference for the initial side used for sound production. Lateralization in sound production has also been studied in other species such as whales (33) and songbirds (34, 35). While we found no evidence for lateralization in this study, future multi-day recordings with identity tracking may yet reveal lateralization on an individual level in *D. cerebrum*.

### The Genus *Danionella* as a Model for Acoustic Communication.

*D. cerebrum*’s native habitat are shallow and turbid waters in Myanmar (16, 17). Male competition in this low-visibility environment likely contributed to the evolution of a specialized mechanism for acoustic communication.

The genus *Danionella* has five species (16) that could be used in comparative studies for sound production. All species of *Danionella* possess a sexually dimorphic fifth rib with the males having a drumming muscle and cartilage (16). Despite these similarities, the sounds produced by *D. dracula* and *cerebrum* are different. *D. dracula* males also produce short pulses, around 2 ms in duration, but the inter-pulse interval distribution is broad and has one peak around 35 ms (36). The duration of the bursts also differs between the two species. *D. cerebrum* can produce bursts that last on the order of seconds, whereas the longest bursts produced by *D. dracula* are only 4 to 6 pulses, with bursts of 2 to 3 pulses being more common (36). The differences in the burst structure may be related to differences in the morphology of their drumming apparatuses as *D. dracula* have a simpler sound-producing organ (20) that lacks the bony flanges and connections of the *os suspensorium* seen in *D. cerebrum* (*SI Appendix*, Fig. S3). The sounds produced by the other species in the *Danionella* genus have not yet been investigated in detail but it will be very interesting to study how the sound production mechanism differs and how these differences may relate to evolutionary adaptation. Together with their lifelong transparency, *Danionella* therefore provide a unique opportunity to study interspecies comparisons of the neural mechanisms underlying vocalizations.

## Methods

### Measuring the Pulse Amplitude.

We placed 4 male wild-type *D. cerebrum* in a 30 × 60 × 30 cm^3^ tank to minimize the effect of sound reflections from the walls of the tank. We placed 5 hydrophones (AS-1, Aquarian Audio) in an array such that they were all 36 mm from the center of the tank, 4 making the corners of a square, and 1 was placed 36 mm below this plane. The audio was amplified with a PA-4 preamplifier (Aquarian Audio) and recorded at a sample rate of 51.2 kHz with a National Instruments 9231 data acquisition card. Pulses were detected in the audio traces using a custom python script. To accurately measure the amplitude, we had to know the distance between the sound source and the hydrophone so we found instances where the timing of the pulse was the same for all of the hydrophones, implying that the pulse was produced by a fish that was in the center of this hydrophone array. The voltage signal from the hydrophones was converted to pressure and then to decibels re 1 μPa.

### High-Speed Video and Analysis.

A small tank was constructed from clear acrylic, with a main compartment measuring 9 cm × 2 cm × 6 cm. The smaller compartment housed an AS-1 hydrophone and was separated from the main compartment with a mesh. This separated the fish from the hydrophone but allowed sound to travel unobstructed. An infra-red LED (CBM-90-IRD-X33-850 nm, Luminus) was mounted so that it illuminated the tank from above, and a diffuser plate was placed on top of the tank so the light would be more evenly distributed. A mirror was placed below the tank and a Ximea CB019MG-LX-X8G3 high-speed video camera was placed to either film the fish from the side or from below using the mirror (Fig. 2*A*).

Groups of 3 to 4 fish, including at least 1 male, were placed inside the tank. Vocalizations were picked up by the hydrophone at a sample rate of 51,200 Hz, which triggered the recording of the video at between 2,000 and 8,000 frames per second. The frame rate limited the dimensions of the field of view of the camera and was initially set lower so more of the tank could be observed. The audio and video recordings were acquired with a National Instruments USB-6363 (BNC) and processed with a custom python script.

To analyze the videos, the vocalizing fish was manually cropped from a single frame and all other frames were registered to this template using opencv-python and scikit-image packages. The difference between adjacent registered frames are plotted in Fig. 2 *B* and *F*. The pixels along the lines indicated in Fig. 2 *B*, *D*, and *E* were plotted for each frame to produce the line scans showing the rapid compression of the swim bladder (Fig. 2 *C*–*E*). Optical flow between two adjacent frames was calculated using functions in scikit-image (37).

### Finite Difference Simulation.

The pressure was calculated following the dimensionless pressure–velocity acoustic equations with a non-reflecting boundary (38) given by

$$\frac{\partial }{\partial t}u=-\nabla p,\frac{\partial p}{\partial t}=-\left(\nabla \cdot u\right),$$

where time *t* is normalized to *l*/*c* (where *l* is the characteristic length measure and *c* the speed of sound), fluid velocity *u* is normalized to *c*, and fluid pressure *p* to *ρc*^2^ (where *ρ* is the mass density of the medium). At each time step, the velocity was calculated in all 3 dimensions and then the pressure was calculated from the velocity.

### Micro-CT Scan and Segmentation.

In accordance with guidelines approved by the regional oversight authority (LAGeSo Berlin) a 13-mo-old male *Danionella* was euthanized with an ice shock and fixed with 4% paraformaldehyde (PFA) in phosphate-buffered saline (PBS) at 4 °C overnight. The next day, the fish was washed in PBS before being stained with 5% phosphomolybdic acid (Sigma Aldrich) solution in PBS at 4 °C overnight. The fish was then washed in PBS for 15 min before embedding in PBS buffered 1% agarose inside a cryo tube. The micro-CT scan was performed at the ANATOMIX beamline at SOLEIL synchrotron by Xploraytion GmbH. The sample was placed into a 40 keV polychromatic (white) X-ray beam. A scan consisted of 3,200 projections that were collected at 10× optical magnification by a digital camera (Orca Flash 4.0 V2) with a sensor pixel size of 6.5 µm at 200 ms exposure time, yielding an effective pixel size of 0.65 µm. The registered data were binned to 2 µm voxel size using linear interpolation.

The components of the drumming apparatus were manually segmented every 20 slices using 3D Slicer and then interpolated using Biomedisa. Smaller details, such as the bi-frication of the inner arm of the os suspensorium described in ref. 16 and Fig. 3 *B* and *C*, were averaged over in the reconstruction but did not affect the model. FIJI ImageJ (39) was used to convert between different file types. The segments were loaded into Blender for cleaning up and rendering.

### Morphological Staining.

A male fish was euthanized with an ice shock and then fixed in 4% PFA at 4 °C overnight. After a short PBS wash, it was then permeabilized with a 1% Triton X-100/0.2% SDS solution at 37 °C over 2 d. The sample was then kept in PBS with 1% Triton X-100 overnight. The following day, the fish was placed in a Phalloidin-iFluor™ 594 working solution composed of 1 μL phalloidin conjugate in 1 mL in PBS solution with 1% bovine serum albumin for 24 h. Finally, the sample was optically cleared before imaging with a 2-photon microscope.

The bone and cartilage were stained with Alizarin Red and Alcian Blue, respectively, in 2 male and 2 female adult fish. Fixation, clearing, and staining protocol was performed as described in ref. 20.

### Differential Gene Expression.

Adult male fish (>6 mo) were euthanized with an ice shock before dissecting both drumming muscles and trunk muscle from 3 to 4 caudal vertebral segments from each fish, for a total of 5 biological replicates for each muscle type pooled from 4 fish each. Total RNA was extracted using a TRIzol Plus RNA Purification Kit (Invitrogen) and the remaining genomic DNA was removed with DNAse I (New England Biolabs). Extracted RNA was poly(A)-selected and sequenced at the MDC/BIH Genomics Platform, Berlin (FacilityID = 1,565). Existing *D. cerebrum* transcriptome annotation (deposited at NCBI as *Danionella translucida* as part of the BioProject PRJNA445947) was updated with the obtained RNA sequencing data using funannotate update pipeline (github.com/nextgenusfs/funannotate). Gene ontology (GO) terms were added using the in-built interproscan annotate routine (40). Read counts were generated using the updated annotation with Salmon in the mapping mode (41). Afterward, the differential expression analysis of drumming and trunk muscle was performed in R using tximport and DESeq2 (42). We used the goseq library to perform GO enrichment tests on differentially expressed genes.

## Supplementary Material

Appendix 01 (PDF)

Dataset S01 (XLSX)

Dataset S02 (AVI)

Dataset S03 (GZ)

Movie S1. **Danionella males vocalize in social contexts** Danionella swimming in their home tank with the spectrogram of the audio below. The males vocalize in the presence of conspecifics.

Movie S2. **Unilateral compression of the swim bladder produces a pulse** A male in the high speed video set up was illuminated from above and recorded from below. The production of a pulse correlates with unilateral compression of the swim bladder. The video was recorded at 8000 frames per second, playback speed 1:100.

Movie S3. **Additional example of high-speed video recording** Another example of a vocalizing male illuminated from above and recorded from below. The fish has been cropped and registered so it remains stationary in the video. The video was recorded at 5000 frames per second, playback speed 1:100.

Movie S4. **Finite difference simulation of pulse in tank** The central blue dot is the source of the pressure pulse, the red dots are the location of the sensors. The edge of the tank is outlined in black and the grayscale indicates the pressure field.

Movie S5. **Vocalizations with a pulse rate of 120 Hz are produced by left-right alternating swim bladder compressions** The fish was cropped, registered and rotated so the swim bladder remained stationary in the video. The pixels along the white line are shown below, highlighting the rapid contraction of the swim bladder and the alternating compressions leading to the 120 Hz pulse rate. It was recorded at 5000 frames per second, playback speed 1:100.

Movie S6. **Vocalizations with a pulse rate of 60 Hz are produced by unilateral compressions of the swim bladder** The fish was cropped, registered and rotated and the pixels along the white line across the swim bladder are displayed below showing that unilateral swim bladder compressions create a 60 Hz pulse rate. The contralateral side remains steady. The video was recorded at 5000 frames per second, playback speed 1:100.

Movie S7. **Movement of the 5th rib correlates with sound production** The tank was illuminated from above and recorded from the side and the vocalizing fish was cropped and registered. The rostral movement of the rib correlates with sound production and the pulse is produced at the peak of the motion, corresponding to the compression of a small area of the swim bladder. The video was recorded at 2000 frames per second, playback speed 1:100.

Movie S8. **Structure anterior to the swim bladder moves towards swim bladder when pulse is produced** The tank was recorded from the side in the brightfield. Movement of a dark structure just anterior to the swim bladder correlates with sound production. Recorded at 5000 frames per second, playback speed 1:100.

Movie S9. **Model of sound production mechanism** A rendering of the sound production mechanism showing the movement of the muscle, rib and cartilage that lead to the compression of the swim bladder.

Audio S1. **Typical vocalization sequence** Example audio recording of Danionella vocalizations.

Audio S2. **Vocalization clip in Fig. 1** The audio clip used in Fig. 1B, C.

## Data Availability

All study data are included in the article and/or supporting information.
