# Supplementary material for: Ultrafast sound production mechanism in one of the smallest vertebrates
Source: Proc Natl Acad Sci U S A. 2024 Feb 26;121(10):e2314017121. doi: 10.1073/pnas.2314017121 (PMC10927587; doi:10.1073/pnas.2314017121)
Supplement: Supplementary file 1 — Appendix 01 (PDF) [file pnas.2314017121.sapp.pdf]

Supplementary Material

Sup. Figure 1: Finite difference simulation of a pulse in an aquarium

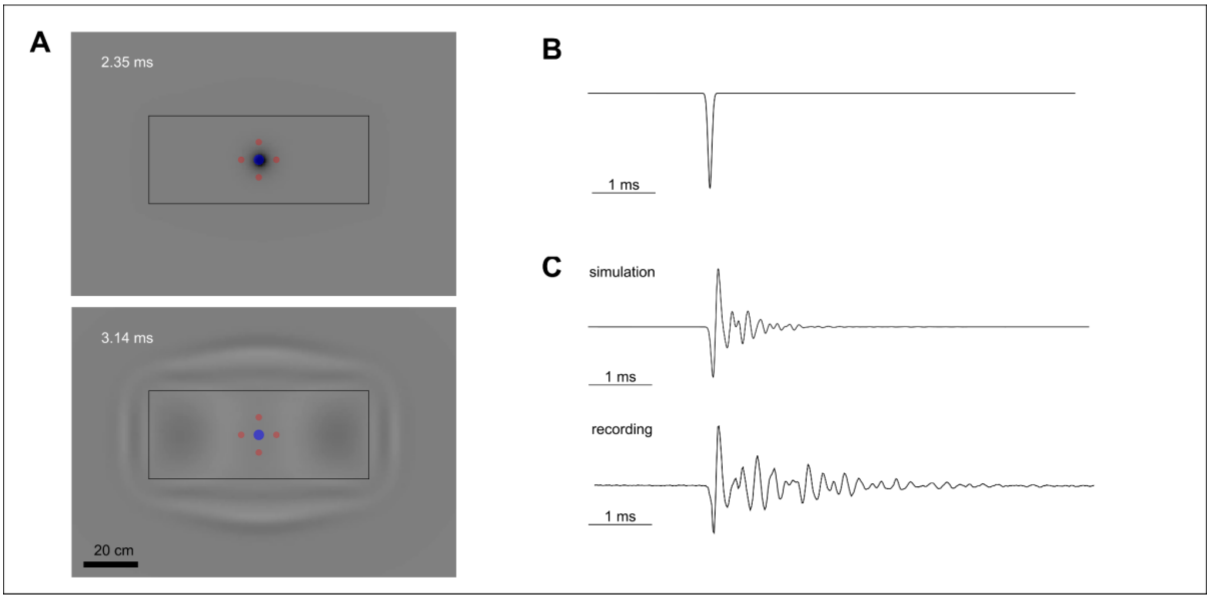

A, Frames from the simulation in the x, y plane. The tank is outlined in black, with the emitter in the center (blue dot) surrounded by 4 detectors (red dots) (Video 4). **B,** The simulated additive pressure pulse from the emitter, modeled as a negative Gaussian with a standard deviation of 40 µs. **C,** Above shows the mean sound detected by the detectors in the finite difference simulation where the oscillations arise from the reflections of the pulse from the boundary between the water and the air. Below is the actual mean sound recorded from hydrophones in a tank of water.

Sup. Figure 2: Calculating the sound pressure level from video recordings

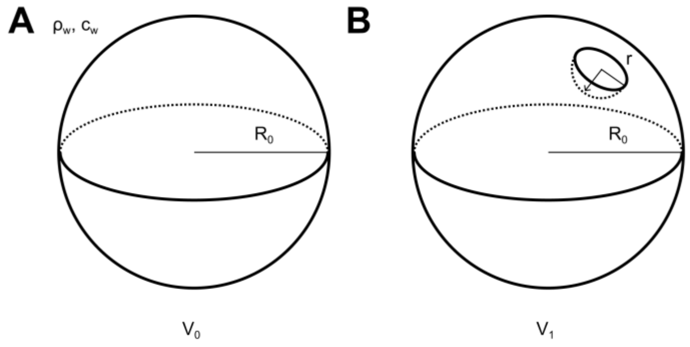

A, The swim bladder was approximated as a sphere with radius R_0 = 0.5 mm (Fig. 2B) and volume $V_0 = \frac{4}{3}\pi {R_0}^3$ surrounded by water with density ρ_w. The speed of sound in water is denoted by c_w = 1500 ms. **B**, The indent caused by the cartilage during sound production was approximated as a hemi-sphere with radius in the range r = 100 µm to 150 µm (Fig. 2C). We estimate that the change in volume occurred over time $\Delta t \approx$ 95 µs (estimate based on the FDTD simulation, in which a Gaussian negative pressure source of ~40 µs S.D. and ~94 µs FWHM was used to fit the recorded traces, see Fig. S1C). Assuming the swim bladder acts as a monopole, this would result in a change in pressure $p = \left| \sqrt[3]{\frac{3}{4\pi}} \frac{\sqrt[3]{V_1} - \sqrt[3]{V_0}}{\Delta t} \frac{\rho_w c_w}{1 + \frac{i}{kR_0}} \right|$. The frequency, f, of the compression itself was estimated to be between 4 kHz and 10 kHz (Fig. S1B) giving the upper and lower bound for the wavenumber $k = \frac{2\pi f}{c_w}$. And thus the estimated sound pressure level 1 m from the center of the swim bladder was calculated to be between 93 and 112 dB, consistent with our empirical measurement of 108 dB (Fig 1F). FDTD: finite difference time domain; FWHM: full width at half maximum.

Sup. Figure 3: Sexual dimorphism in structures related to the drumming apparatus

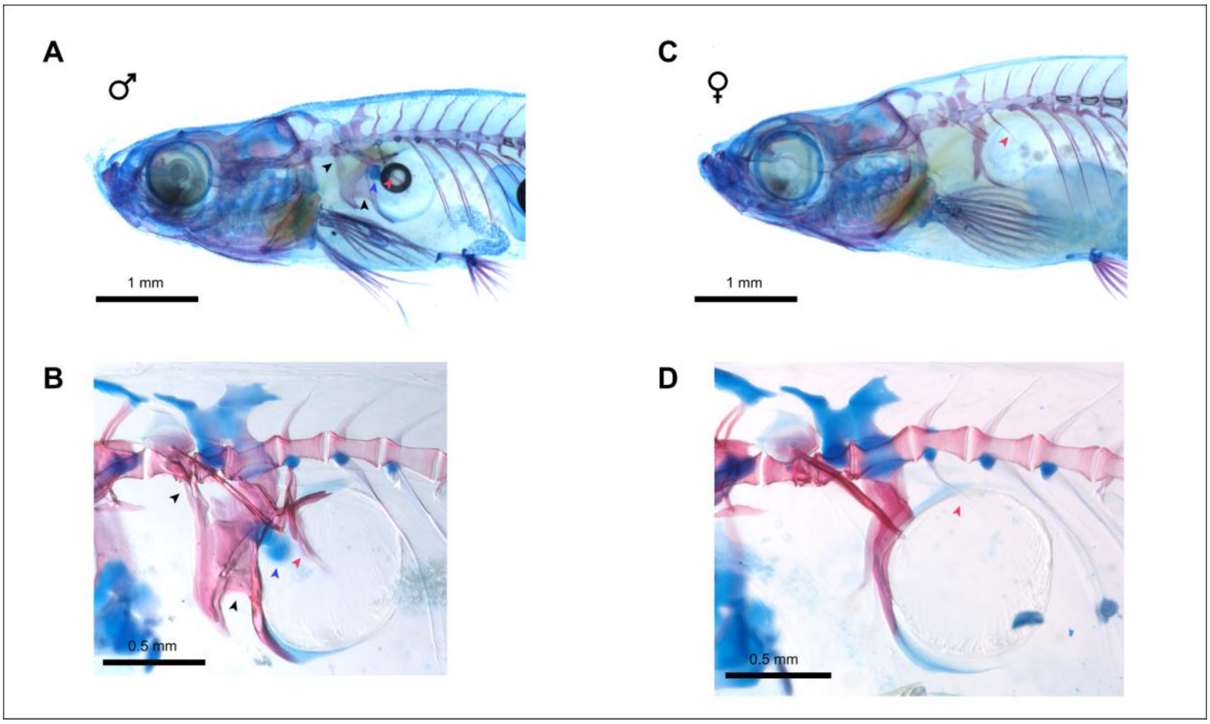

A, Male and **C,** female *Danionella cerebrum* in which bones are stained with Alizarin Red and cartilage with Alcian Blue. In the males, the outer and inner arms of the *os suspensorium* are connected to each other by flanges of bone, and the outer arm is connected by a bone extension to the lateral process of the second vertebra (black arrowheads). In the males the inner arm also covers the anterodorsal face of the swim bladder. These bone connections and extensions are absent in the females. The fifth rib is heavily ossified in the males (red arrowhead) and the globular piece of drumming cartilage (blue arrowhead) of males is completely absent in females. **B, D,** close ups of the relevant region in a male and female respectively (different individuals).

Sup. Figure 4: Genes associated with mitochondria are overexpressed in the drumming muscle

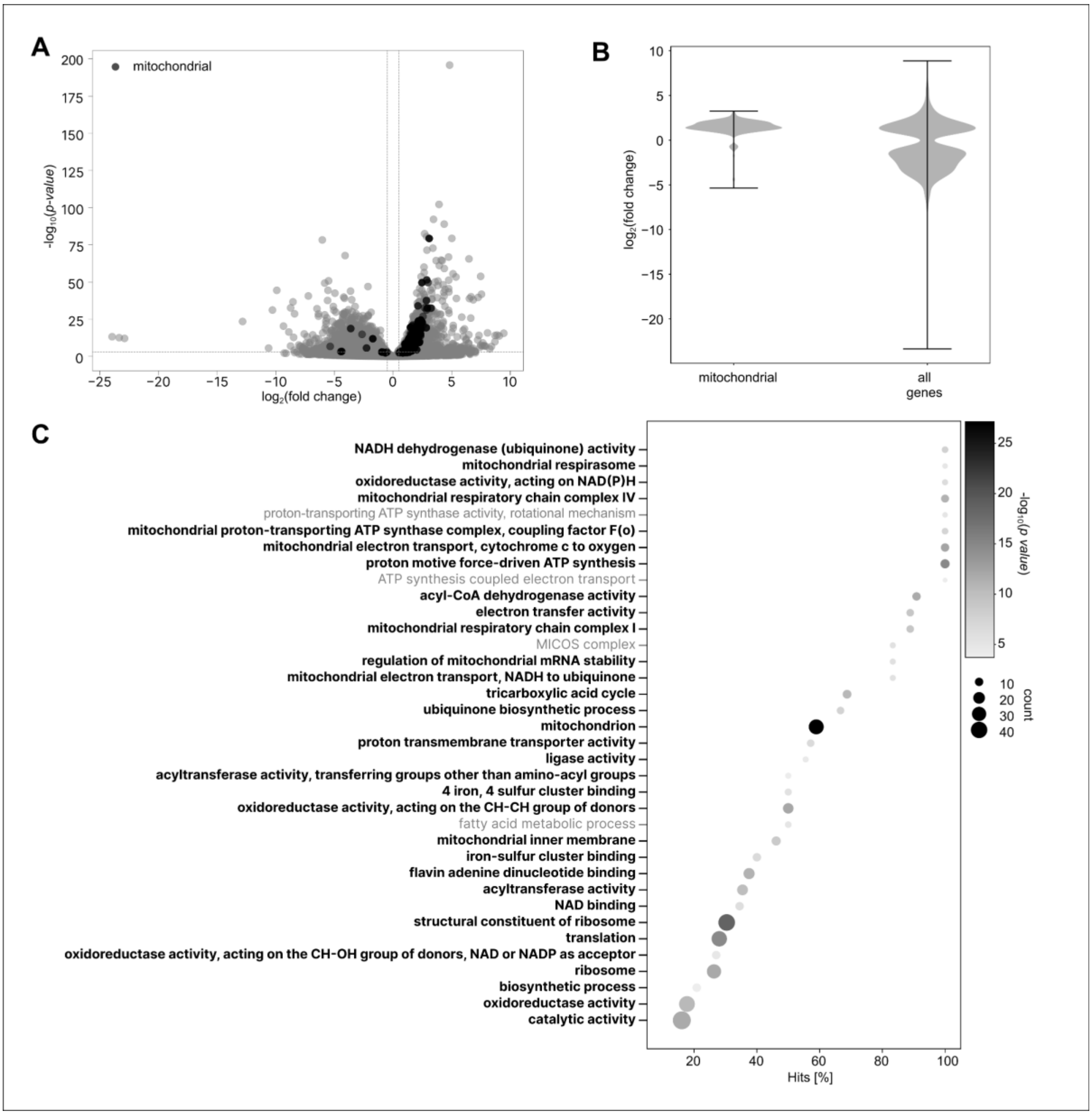

A, A volcano plot of the log p value against the log2 fold change of all of the differentially expressed genes. The mitochondrial genes, plotted in black, are vastly overexpressed in the drumming muscle compared to the trunk muscle in the tail. **B,** Violin plots of the log2 fold change of the significant genes in A. 94% of genes associated with mitochondria had a positive log2 fold change value and so were over expressed in the drumming muscle in contrast to 43% of the genes. **C**, The categories (Gene ontology terms) with the most significant *p-values* were arranged according to the percentage of genes within these categories that were overexpressed in the drumming muscle. The categories associated with mitochondria are in bold. “Count” refers to the total number of genes within this category.

Supplementary file legends

Video 1: Danionella males vocalize in social contexts

Danionella swimming in their home tank with the spectrogram of the audio below. The males vocalize in the presence of conspecifics.

Video 2: Unilateral compression of the swim bladder produces a pulse

A male in the high speed video set up was illuminated from above and recorded from below. The production of a pulse correlates with unilateral compression of the swim bladder. The video was recorded at 8000 frames per second, playback speed 1:100.

Video 3: Additional example of high-speed video recording

Another example of a vocalizing male illuminated from above and recorded from below. The fish has been cropped and registered so it remains stationary in the video. The video was recorded at 5000 frames per second, playback speed 1:100.

Video 4: Finite difference simulation of pulse in tank

The central blue dot is the source of the pressure pulse, the red dots are the location of the sensors. The edge of the tank is outlined in black and the grayscale indicates the pressure field.

Video 5: Vocalizations with a pulse rate of 120 Hz are produced by left-right alternating swim bladder compressions

The fish was cropped, registered and rotated so the swim bladder remained stationary in the video. The pixels along the white line are shown below, highlighting the rapid contraction of the swim bladder and the alternating compressions leading to the 120 Hz pulse rate. It was recorded at 5000 frames per second, playback speed 1:100.

Video 6: Vocalizations with a pulse rate of 60 Hz are produced by unilateral compressions of the swim bladder

The fish was cropped, registered and rotated and the pixels along the white line across the swim bladder are displayed below showing that unilateral swim bladder compressions create a 60 Hz pulse rate. The contralateral side remains steady. The video was recorded at 5000 frames per second, playback speed 1:100.

Video 7: Movement of the 5th rib correlates with sound production

The tank was illuminated from above and recorded from the side and the vocalizing fish was cropped and registered. The rostral movement of the rib correlates with sound production and the pulse is produced at the peak of the motion, corresponding to the compression of a small area of the swim bladder. The video was recorded at 2000 frames per second, playback speed 1:100.

Video 8: Structure anterior to the swim bladder moves towards swim bladder when pulse is produced

The tank was recorded from the side in the brightfield. Movement of a dark structure just anterior to the swim bladder correlates with sound production. Recorded at 5000 frames per second, playback speed 1:100.

Video 9: Model of sound production mechanism

A rendering of the sound production mechanism showing the movement of the muscle, rib and cartilage that lead to the compression of the swim bladder.

Audio 1: Typical vocalization sequence
Example audio recording of Danionella vocalizations.

Audio 2: Vocalization clip in Fig. 1

The audio clip used in Fig. 1B, C.

Supplemental Dataset 1: Sound pressure level, length and weight for animals in Fig 1F

Supplemental Dataset 2: micro CT of drumming apparatus (see Figure 3)

Supplemental Dataset 3: 3D mesh STL file of the drumming apparatus (see Figure 3)
